# SOCIAL RELATIONS AND OLDER MIGRANTS

**DOI:** 10.1093/geroni/igad104.1634

**Published:** 2023-12-21

**Authors:** Kristine Ajrouch, Toni Antonucci, Rita Hu

**Affiliations:** Eastern Michigan University, Ypsilanti, Michigan, United States; University of Michigan, Ann Arbor, Michigan, United States; University of Michigan, Ann Arbor, Michigan, United States

## Abstract

This paper elaborates on how the key life course tenets of timing and context represent important factors that influence older adult social relations following the act of migration. The timing of migration may yield specific social relations. For instance, migrating later in life is likely to result in more homogenous and restricted networks compared to migrating as a young adult. Reasons for migration (political instability, family reunification, or socio-economic aspirations) as well as host country factors such as immigrant policies and receptivity infer important contexts in which social relations emerge. The process of movement from one country to another, and the circumstances around those movements, frame the migrant experience and its effects on social relations. Beyond timing and context, there are also demographic and cultural factors that influence the experience. For instance, gender, race and SES indicate social positions in systems of stratification that can influence and structure the people with whom one has ties. Further, cultural factors such as country of origin and language not only signal the ease with which host society incorporation occurs, but also inform the acculturation and meaning-making processes upon which the formation and function of social relations rest. In sum, the concepts found in the life course perspective provide a useful starting point to demonstrate that the effects of migration on older adults’ social relations occur within various influential contexts.

